# Probing Oxygen-to-Hydrogen Peroxide Electro-Conversion at Electrocatalysts Derived from Polyaniline

**DOI:** 10.3390/polym14030607

**Published:** 2022-02-04

**Authors:** Yaovi Holade, Sarra Knani, Marie-Agnès Lacour, Julien Cambedouzou, Sophie Tingry, Teko W. Napporn, David Cornu

**Affiliations:** 1 Institut Européen des Membranes, IEM, UMR 5635, Univ Montpellier, ENSCM, CNRS, 34090 Montpellier, France; sarra.knani@umontpellier.fr (S.K.); julien.cambedouzou@enscm.fr (J.C.); sophie.tingry@umontpellier.fr (S.T.); david.cornu@enscm.fr (D.C.); 2 ChemLab, ENSCM, 34296 Montpellier, France; marie-agnes.lacour@enscm.fr; 3 IC2MP, Université de Poitiers, UMR-CNRS 7285, CEDEX 9, 86073 Poitiers, France; teko.napporn@univ-poitiers.fr

**Keywords:** conducting polymer, polyaniline, electrocatalysis, hydrogen peroxide, oxygen reduction reaction, electrosynthesis

## Abstract

Hydrogen peroxide (H_2_O_2_) is a key chemical for many industrial applications, yet it is primarily produced by the energy-intensive anthraquinone process. As part of the Power-to-X scenario of electrosynthesis, the controlled oxygen reduction reaction (ORR) can enable the decentralized and renewable production of H_2_O_2_. We have previously demonstrated that self-supported electrocatalytic materials derived from polyaniline by chemical oxidative polymerization have shown promising activity for the reduction of H_2_O to H_2_ in alkaline media. Herein, we interrogate whether such materials could also catalyze the electro-conversion of O_2_-to-H_2_O_2_ in an alkaline medium by means of a selective two-electron pathway of ORR. To probe such a hypothesis, nine sets of polyaniline-based materials were synthesized by controlling the polymerization of aniline in the presence or not of nickel (+II) and cobalt (+II), which was followed by thermal treatment under air and inert gas. The selectivity and faradaic efficiency were evaluated by complementary electroanalytical methods of rotating ring-disk electrode (RRDE) and electrolysis combined with spectrophotometry. It was found that the presence of cobalt species inhibits the performance. The selectivity towards H_2_O_2_ was 65–80% for polyaniline and nickel-modified polyaniline. The production rate was 974 ± 83, 1057 ± 64 and 1042 ± 74 µmol_H2O2_ h^−1^ for calcined polyaniline, calcined nickel-modified polyaniline and Vulcan XC 72R (state-of-the-art electrocatalyst), respectively, which corresponds to 487 ± 42, 529 ± 32 and 521 ± 37 mol kg^−1^_cat_ h^−1^ (122 ± 10, 132 ± 8 and 130 ± 9 mol kg^−1^_cat_ cm^−2^) for faradaic efficiencies of 58–78%.

## 1. Introduction

Hydrogen peroxide (H_2_O_2_) is one of the most important chemicals in the chemical and medical industries. Its industrial production largely depends on the anthraquinone process, an energy-intensive process that involves the hydrogenation of 2-alkylanthraquinone using expensive palladium catalysts and generates numerous by-products [1,2]. Indeed, to address the high and increasing demand (application for disinfection, textile bleaching, wastewater treatment and renewable energy storage), the four possible methods to produce H_2_O_2_ include the traditional anthraquinone process, direct synthesis, photocatalysis and electrocatalysis [3,4,5]. As an alternative to the anthraquinone process, the direct reaction (H_2_ + O_2_ → H_2_O_2_) is conceptually the simplest method, but it requires high pressure and finding H_2_ that is not yet produced in a decarbonized way. The photocatalysis that provides the required energy for the direct method by the solar power is contingent on climatic conditions, so it may not be the sole answer. The electrocatalytic [3,6,7,8,9,10] (or even photoelectrocatalytic [11,12]) pathway, the so-called Power-to-X approach, is thus seen as a promising option where electrical energy from any source (ideally renewable) would allow the intelligent and elegant production of hydrogen peroxide from naturally available reactants, either by the partial oxidation of water (2H_2_O → H_2_O_2_ + 2H^+^ + 2e^−^) or the partial reduction of atmospheric oxygen (O_2_ + 2H^+^ + 2e^−^ → H_2_O_2_). For that, we need highly selective electrocatalysts to circumvent the four-electron pathway that reduces the faradaic efficiency (portion of the used electrical energy to make a targeted electron transfer reaction).

For environmentally friendly chemistry, the electrocatalytic pathway of reducing O_2_ from air under alkaline media (O_2_ + H_2_O + 2e^−^ → HO_2_^−^ + HO^−^, E° = 0.76 V vs. RHE, reversible hydrogen electrode) at ambient conditions is increasingly being considered, ideally by using noble metal-free electrocatalysts for chemical stability and cost reduction reasons [13,14]. To achieve such an alternative, we would need to develop a material that would not push the reduction very far, O_2_ + 2H_2_O + 4e^−^ → 4HO^−^, E° = 1.23 V_RHE_. As reported in Ref. [3], a selectivity of 60–90% is reported for oxygen-to-hydrogen peroxide using the electroanalytical method of rotating ring-disk electrode (RRDE), which is associated with a faradaic efficiency surpassing 80% in bulk electrolysis systems (record production rate of 3660 mol_H2O2_ kg_cat_^−1^ cm^−2^ (3.4 mmol_H2O2_ cm^−2^ h^−1^) for a faradaic efficiency of 90% at a cell voltage of 0.6 V (current density of 200 mA cm^−2^) [8]).

While polyaniline-based materials were used to engineer electrocatalysts for ORR, few studies focused on controlling the selectivity towards the two-electron pathway to produce hydrogen peroxide [15,16,17]. Rabl et al. [17] found that the electrochemically grown conducting polymers of polyaniline (PANI) and polypyrrole onto carbon paper electrodes can efficiently perform the oxygen-to-hydrogen peroxide electro-conversion in a wide window of pH. Quílez-Bermejo et al. [18] reported PANI-derived N-doped mesoporous carbons for ORR in alkaline media, but the depth of the reaction was not scrutinized to evaluate whether a four-electron pathway was followed or whether hydrogen peroxide is the main product. Our recent and systematic studies [19,20] showed that the synthesis conditions and the post-synthesis thermal treatment of raw PANI under various conditions can produce different types of materials with different electrocatalytic behaviors. However, we currently lack knowledge about the effect of chemical and thermal modifications of PANI on the performance in hydrogen peroxide production of a selective two-electron pathway of ORR. To fill this gap in the knowledge, the aim of the present work is to conduct a case study on the potentiality of oxygen-to-hydrogen peroxide electro-conversion at electrocatalysts made of PANI modified or not by nickel and cobalt species and having undergone or not a thermal treatment. The study was conducted by combining RRDE and bulk electrolysis in alkaline media.

## 2. Experimental

### 2.1. Materials and Chemicals

Potassium hydroxide (KOH, 99.98% (trace metal basis), Acros Organics), nickel (II) nitrate hexahydrate (Ni(NO_3_)_2_·6H_2_O, 99%, Acros Organics, Illkirch Cedex, France), hydrochloric acid (HCl, 37%, VWR, Rosny-sous-Bois, France), cobalt (II) nitrate hexahydrate (Co(NO_3_)_2_·6H_2_O, ACS, 98.0–102.0%, Alfer Aesar, Havehill, MA, USA), aniline (ANI, 100%, Alfa Aesar), ammonium persulfate ((NH_4_)_2_S_2_O_8_, 98%, Merck, Kenilworth, NJ, USA), isopropanol (iPrOH, 99.5%, Sigma Aldrich, Saint Louis, MO, USA) and Nafion^®^ suspension (5 wt%, Sigma Aldrich) were used as received. Commercial carbon black, type Vulcan XC72R, was provided by Cabot Corporation (Europe, Middle East & Africa; SIA Cabot Latvia, Riga, Latvia) and thermally activated before use by the procedure of Ref. [21]. A carbon paper electrode (AvCarb MGL370, 370 μm thickness) was purchased from Fuel Cell Earth LL (USA) and washed with iPrOH prior to use. Ultrapure water was produced from a Milli-Q Millipore source (18.2 MΩ cm at 20 °C). Nitrogen (N_2_) and oxygen (O_2_) were ultrapure (Air Liquide, Paris, France).

### 2.2. Synthesis of Polyaniline-Based Materials by Oxidative Polymerization and Thermal Treatments

A line up of nine polyaniline-derived materials were prepared by our optimized method of the oxidative polymerization of aniline, triggered by APS in an acidic medium as reported in Ref. [20]. The starting mixture was an acidified (0.5 M HCl) solution of 0.4 M ANI in the absence and presence of 0.181 M of Ni(NO_3_)_2_·6H_2_O or Co(NO_3_)_2_·6H_2_O at 5 °C. This mixture was titrated by an acidified (0.5 M HCl) solution of APS (0.2 M, 100 mL) at five milliliters per minute using a two-syringe infusion pump (KD Scientific, Holliston, MA, USA). After 13 h of polymerization, the solvent was removed using a rotavap before drying at 80 °C overnight in an oven to recover a solid polymer product. At this stage, the three materials were referred to as PANI, PANI-Ni and PANI-Co. Next, these materials underwent a thermal stabilization step and/or reticulation of the polymer (TS) under air at 120 °C h^−1^ up to an optimized temperature of 350 °C for 2 h [20]. The achieved materials are hereinafter termed as PANI-TS, PANI-Ni-ST and PANI-Co-TS. Lastly, the former samples were treated under inert gas (N_2_) at 300 °C h^−1^ up to a dwell of 50 °C (1 h) and slowed down to 120 °C h^−1^ toward the optimized temperature of 900 °C for 6 h [20]. The obtained materials were referred to as PANI-TS-TC, PANI-Ni-TS-TC and PANI-Co-TS-TC.

### 2.3. Physicochemical Characterization

We note that the motivation of the present work was to interrogate the electrocatalytic ability of the previous materials that were extensively characterized by XPS, BET, ICP, CNHSO, Raman spectroscopy and FTIRS, as reported in Refs. [19,20]. Herein, we included few characterizations of the nine synthesized samples by scanning electron microscopy (SEM), energy-dispersive X-ray spectroscopy (EDX) and power diffraction X-ray (XRD). SEM was conducted on a Hitachi S-4800 FEG. EDX was performed by using a ZEISS EVOHD 15 microscope. XRD was performed by using a PANalytical Xpert-PRO diffractometer (40 kV, 20 mA) equipped with a copper anode (λ(CuKα) = 1.54 Å) in Bragg–Brentano mode.

### 2.4. Electrochemical Measurements, Bulk Electrolysis and UV-Vis Assays

An AUTOLAB PGSTAT128N bipotentiostat-galvanostat (Metrohm, Barendrecht, Netherlands) equipped with a linear SCAN250 module (Analog scan generator, enables the true linear scan from 10 mV s^−1^ to 250 kV s^−1^) was used for the electrochemical measurements. The used methods were cyclic voltammetry (CV), linear sweep voltammetry (LSV) and chronoamperometry (CA). RRDE setup (Metrohm, Netherlands) was composed of a glassy carbon disk (5 mm diameter, 0.196 cm^2^) and a Pt ring (0.072 cm^2^). RRDE was manually polished by alumina slurries of increasing particles size (3, 1 and 0.05 μm) and washed with ultrapure water by sonication. At the same time, a catalytic ink was prepared by ultrasonically mixing (water batch) 130 µL of ultrapure water, 50 µL of isopropanol, 20 µL of Nafion^®^ suspension and 5 mg of electrocatalyst. After drying the RRDE, a volume of 4 µL was deposited onto the disk to yield 0.5 mg_cat_ cm^−2^. Then, the rotator speed was increased to 400 rpm to not only facilitate the solvents evaporation under ambient conditions, but also to obtain an homogeneous thin-film of the catalytic layer and repeatable/reproducible experiments [22,23,24,25]. After degassing the electrolyte (1 M KOH solution) by N_2_ for 20 min, the CV of the blank was recorded in a three-electrode setup where the working electrode was the RRDE disk, the counter electrode was a large surface-area glassy carbon plate and the reference electrode was Ag/AgCl (calibrated versus RHE in H_2_-saturated electrolyte: E_RHE_ − E_Ag/AgCl_ = 0.964 V). For ORR, the electrolyte was saturated by O_2_ for 20 min and the potential of the disk was scanned in the cathodic direction from the open circuit potential (OCP) to 0.1 V_RHE_. The potential of the RRDE ring was poised at E_ring_ = 1.2 V_RHE_ to oxidize any hydrogen peroxide product from ORR back to O_2_ and lead to a quantitative metric by the ratio of between the ring current and that of the disk, I_D_/I_R_. We note that the predominant form is hydroperoxide anion HO_2_^−^ because of the electrolyte’s pH of ~14 and p*K*a(H_2_O_2_/HO_2_^−^) = 11.75.

The quantitative analysis by bulk electrolysis and UV-vis assays were described in Ref. [9]. Typically, the two compartments of a H-type cell were separated by a hydroxide anion exchange membrane and each contained 60 mL of 1 M KOH at room temperature (22 ± 2 °C). The cathodic compartment saturated by O_2_ held a carbon paper electrode (AvCarb MGL370, 370 μm thickness, 1 cm × 2 cm) coated with the above catalytic ink at 0.5 mg_cat_ cm^−2^ (both sides), and its potential was poised at 0.6 V_RHE_ (iR-uncorrected) for 1 h. Aliquots were sequentially taken for UV-vis assays using the UviLine Connect 940 spectrophotometer (Spectralab software). Each solution was treated with an equivalent excess of H_2_SO_4_ (at 0.34 mol per L to generate H_2_O_2_ species: HO_2_^−^_(aq)_ + H^+^_(aq)_ → H_2_O_2_, pKa = 11.75) and potassium titanium (IV) oxalate (at 50 g per L to form a colored indicator with H_2_O_2_). After preliminary tests in a full spectrum configuration (300–900 nm), the absorbance of the yellow pertitanic acid complex between hydrogen peroxide and potassium titanium oxalate was measured at 390 nm [9,26,27,28,29] in the kinetic configuration.

## 3. Results and Discussion

### 3.1. Electrochemical Analysis

**Figure 1:**
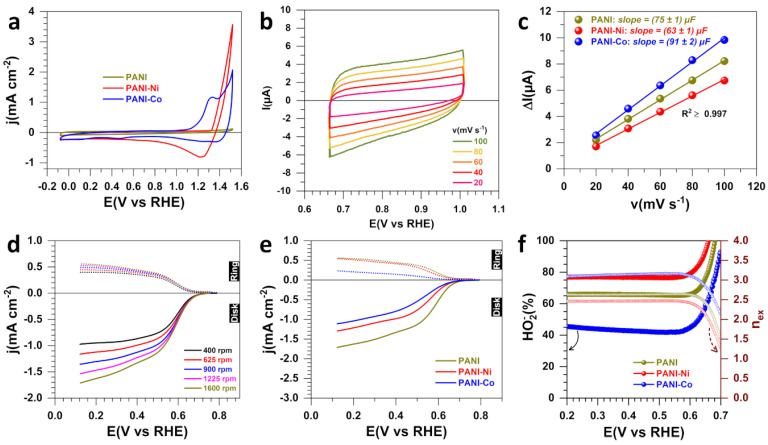
Electrochemical characterization for the materials obtained after polymerization (5 °C, 13 h). (**a**) iR-drop uncorrected CV (N_2_-saturated 1 M KOH, 25 °C, 100 mV s^−1^, 0 rpm). (**b**) iR-drop uncorrected CV of PANI-Ni (N_2_-saturated 1 M KOH, 25 °C, 0 rpm) for determining ECSA. (**c**) Capacitive current (ΔI_a_ = I_a_−I_c_) vs. scan rate at E(V_RHE_) = 0.8 (PANI), 1.0 (PANI-Ni) and 0.8 (PANI-Co). (**d**) iR-drop uncorrected LSV of ORR for PANI at different speeds of RRDE (O_2_-saturated 1 M KOH, 25 °C, 5 mV s^−1^, E_ring_ = 1.2 V_RHE_). (**e**) iR-drop uncorrected LSV of ORR at RRDE (O_2_-saturated 1 M KOH, 25 °C, 5 mV s^−1^, 1600 rpm, E_ring_ = 1.2 V_RHE_). (**f**) HO_2_^−^% (left) and number of transferred electrons per molecule of O_2_ (right *y*-axis) from panel (**e**).

To better understand the electrochemical behaviors of the nine synthesized materials, the CV of each electrode was recorded in N_2_-saturated 1 M KOH at 25 °C and 100 mV s^−1^ (0 rpm). We chose not to apply ohmic drop correction, even though the ohmic resistance was in the range of 5–6 Ω as determined by the electrochemical impedance spectroscopy (EIS), indicating an ohmic drop in potential of 25–30 mV at an absolute current of 5 mA. Figure 1a shows the blank CV profiles for the three materials after the oxidative chemical polymerization. Compared to acidic media where characteristic redox processes for the oxidation states of PANI can be observed [19,30], in the alkaline environment, there is no major oxidation or reduction peak of electron transfer. When the polymerization was conducted in the presence of nickel or cobalt species (Ni^2+^ and Co^2+^), the redox processes associated with the oxy–hydroxide transitions can be observed between 1.0 V_RHE_ and 1.5 V_RHE_ [M(OH)_2_ + OH^−^ = MOOH + H_2_O + e^−^, M = Ni, Co].

**Figure 2:**
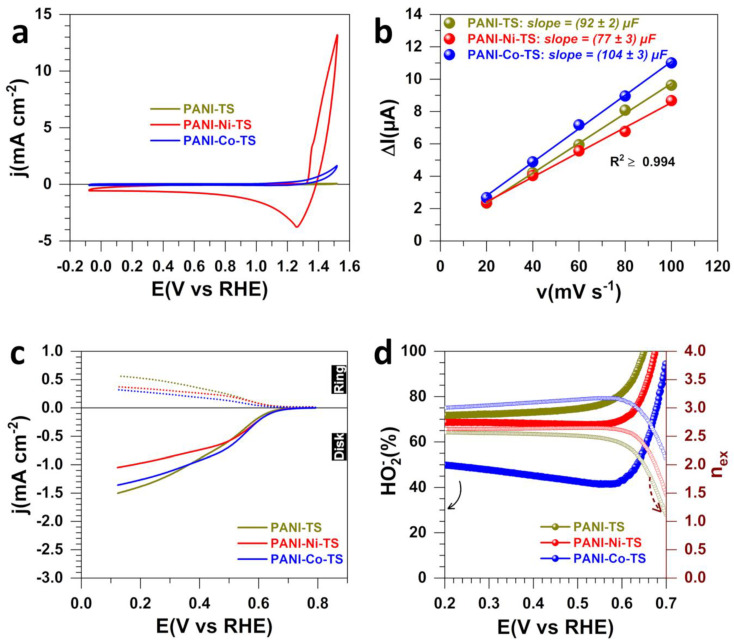
Electrochemical characterization for the materials obtained after polymerization (5 °C, 13 h) and stabilization (air, 350 °C, 2 h). (**a**) iR-drop uncorrected CV (N_2_-saturated 1 M KOH, 25 °C, 100 mV s^−1^, 0 rpm). (**b**) Capacitive current (ΔI_a_ = I_a_−I_c_) vs. scan rate at E(V_RHE_) = 0.8 (PANI-TS), 1.2 (PANI-Ni-TS) and 0.7 (PANI-Co-TS). (**c**) iR-drop uncorrected LSV of ORR at RRDE (O_2_-saturated 1 M KOH, 25 °C, 5 mV s^−1^, 1600 rpm, E_ring_ = 1.2 V_RHE_). (**d**) HO_2_^−^% (left) and number of transferred electrons per molecule of O_2_ (right *y*-axis) from panel (**c**).

We next used the electroanalytical method of the electrochemical double-layer capacitance to determine whether the introduced Ni(+II) and Co(+II) species modified the electrochemically active surface area (ECSA) according to the relationship ECSA = C_dl_/C_s_, where C_dl_ is the capacitance and C_s_ is the average specific capacitance (about 40 μF cm^−2^) [31,32]. To this end, the voltammograms were recorded in the capacitive region at different scan rates. Figure 2b shows the results for PANI, while those of PANI-Ni and PANI-Co are reported in Figure S2. The plots of the capacitive current (ΔI_a_ = I_a_−I_c_) versus the scan rate at a given electrode potential (E(V_RHE_) = 0.8, 1.0 and 0.8 for PANI, PANI-Ni and PANI-Co, respectively) display a linear trend where the slope is twice the capacitance. The trend is PANI-NI (C_dl_ = 63 ± 1 µF) < PANI (C_dl_ = 75 ± 1 µF) < PANI-Co (C_dl_ = 91 ± 2 µF), which indicates a modification in the number of the available electrochemical active sites. The next question is whether all these active sites are in favor of a better hydrogen peroxide production by selective O_2_ reduction. It is known that the selectivity toward hydrogen peroxide production requires O_2_ molecules to be perpendicularly adsorbed by a single oxygen atom on the catalytic surface (a parallel adsorption will trigger the oxygen-oxygen bond cleavage), which can be achieved by tuning the inter-distance between active sites and the heterogeneity of the catalytic surface [9,33,34,35]. In the following, we will briefly explain the basics of the used electroanalytical method to track the depth of the ORR on-line.

During ORR in alkaline media, O_2_ molecule can receive either four or two electrons to produce OH^−^ according to the net Equation (1) or to yield HO_2_^−^ according to Equation (2). The produced hydroperoxide anion can then be reduced into OH^−^ according to Equation (3) or be stable as the final product. For the latter case, it can be oxidized at the ring of an RRDE setup [36]. Hence, by employing a RRDE setup [36,37], it is possible to derive electroanalytical relationships that link the transferred number of electrons per molecule of O_2_ (n_ex_) and the amount of hydrogen peroxide to the current of the disk (I_D_), the current of the ring (I_R_) and the collection efficiency (N: defined as the fraction of the species formed at the disk that arrive at the ring and react there [36]). Because the value of the collection efficiency is specific to the geometry of the RRDE and does not depend on the studied redox reaction, we have determined it by employing the simple redox probe [Fe((CN)_6_]^3−^/[Fe(CN)_6_]^4−^ using a four-electrode potentiostat where both potentials of the disk and the ring are controlled with respect to the reference electrode independently. The results are reported in Figure S1 and give an average collection efficiency of 24.9 ± 0.3%, which is in line with the manufacturer’s value of 24.9%.

O_2_ + 2H_2_O + 4e^−^ → 4HO^−^ (1)

O_2_ + H_2_O + 2e^−^ → HO_2_^−^ + HO^−^ (2)

HO_2_^−^ + H_2_O + 2e^−^ → 3HO^−^ (3)

Let I_1_, I_2_, and I_3_ be the currents corresponding to these three electrochemical processes of ORR at the disk of RRDE (I_D_ = I_1_ + I_2_ + I_3_). I_1_, I_2_ and I_3_ have the same sign (counted either as positive or negative depending on the used convention to plot the current-potential curves [38]), meaning that mathematic rules do apply. According to the second law of Faraday, the total number of O_2_ molecules participating in ORR is given by Equation (4) below.

(4) $$N(O_{2})=\left[{\left(n(O_{2})\right)}_{involved\;in\;4-electron\;process}+{\left(n(O_{2})\right)}_{involved\;in\;2-electron\;process}\right]N_{A}=\left|I_{1}+2I_{2}\right|\frac{tN_{A}}{4F}$$

In Equation (4), *t* = duration, *N_A_* = Avogadro constant, *F* = Faraday constant, and |*I*| = absolute value of the current. By taking into account the conservation of matter (Lavoisier), the total transferred number of electrons and the total number of produced hydroperoxide anion HO_2_^−^ molecules can be readily evaluated by Equations (5) and (6), respectively.

(5) $$N(e^{-})=\left|I_{1}+I_{2}+I_{3}\right|\frac{tN_{A}}{F}$$

(6) $$N(HO_{2}^{-})=\left[{\left(n(HO_{2}^{-})\right)}_{involved\;in\;Eq.\;S2}-{\left(n(HO_{2}^{-})\right)}_{involved\;in\;\mathrm{Eq}.\;S3}\right]N_{A}=\left|I_{2}-I_{3}\right|\frac{tN_{A}}{2F}$$

Considering that the collection efficiency is given by Equation (7) and that the disk current is I_D_ = I_1_ + I_2_ + I_3_, the searched overall exchanged number of electrons per molecule of O_2_ is expressed by Equation (8) and the fraction of the produced hydroperoxide anion HO_2_^−^ per molecule of O_2_ is given by Equation (9). We note that the quantity HO_2_^−^(%) is a selectivity metric, not the faradaic efficiency.

(7) $$N=\left|\frac{I_{R}}{{\left(I_{D,total}\right)}_{involving\;HO_{2}^{-}\;species}}\right|=\left|\frac{I_{R}}{I_{2}-I_{3}}\right|\Rightarrow \left|I_{R}\right|=\left|I_{2}-I_{3}\right|N$$

(8) $$n_{ex}=\frac{N(e^{-})}{N(O_{2})}=4\frac{I_{1}+I_{2}+I_{3}}{I_{1}+2I_{2}}=4\frac{\left|I_{1}+I_{2}+I_{3}\right|}{\left|I_{1}+I_{2}+I_{3}\right|+\left|I_{2}-I_{3}\right|}=4\frac{1}{1+\left|\frac{I_{R}}{NI_{D}}\right|}=\frac{4}{1-\frac{I_{R}}{NI_{D}}}$$

(9) $$HO_{2}^{-}(\%)=100\frac{N(HO_{2}^{-})}{N(O_{2})}=200\frac{\left|I_{2}-I_{3}\right|}{\left|I_{1}+2I_{2}\right|}=200\frac{\left|I_{2}-I_{3}\right|}{\left|I_{1}+I_{2}+I_{3}\right|+\left|I_{2}-I_{3}\right|}=\frac{200}{1-\frac{NI_{D}}{I_{R}}}$$

We note that I_D_ and I_R_ have opposite signs (I_D_ is counted either as positive or negative depending on the used convention to plot the current-potential curves [38]). Therefore, the ratio I_D_/I_R_ is algebraically a negative number, which justifies the sign (−) in the absence of the absolute value to avoid the frequently made mistake in the literature where people use the sign (+). Figure 1d shows the representative current–potential profiles of the disk and the ring at different speeds of RRDE for PANI. The corresponding percentage of HO_2_^−^ and the number of transferred electrons per molecule of O_2_ from this iR-drop uncorrected LSV of ORR are reported in Figure S3. For the three materials obtained from the polymerization step, the comparison at 1600 rpm is shown in Figure 1e for LSV and in Figure 1f for HO_2_^−^, along with the number of transferred electrons per molecule of O_2_. The LSV shows that ORR starts at a potential close to the expected potential for the process O_2_ + H_2_O + 2e^−^ → HO_2_^−^ + HO^−^, E° = 0.76 V_RHE_. It is worth noting, however, that this could also correspond to the other process with four electrons (O_2_ + 2H_2_O + 4e^−^ → 4HO^−^, E° = 1.23 V_RHE_). Indeed, the starting potential in the case of ORR is not a fair descriptor because ORR has slow kinetics and not all of the species of the redox couple O_2_/HO_2_^−^ are present in the beginning to satisfy the condition of Nernst thermodynamics. Only quantitative analysis can provide insightful information.

While the amount of HO_2_^−^ and the total transferred electrons per molecule of O_2_ (*n*_ex_) were evaluated online by Equations (8) and (9), the Levich relationship of Equation (10) can be used to evaluate *n*_ex_ [37,39] when the plateau for limiting current density is well-defined. We note that the validity of Levich’s law is subject to the following two requirements:

(i) The existence of a mass transport process that is the rate-determining step (rds),
(ii) The reaction is of a first order reaction with respect to the electroreactive species (O_2_).

The kinetic current density *j*_k_ as the absolute activity can be derived from Equation (11) before the conversion into the mass activity *j*_k_(A mg^−1^) by the division of the absolute value by the electrocatalyst loading (mg_cat_ cm^−2^_geometric_) or specific kinetic current density *j*_k_(mA cm^−2^_ECSA_) [36,37].

(10) $$j_{\lim}=n_{ex}\times B\times \Omega^{\frac{1}{2}},\;\;\;\;\;B=0.201FC\upsilon^{-\frac{1}{6}}D^{\frac{2}{3}}$$

(11) $$\frac{1}{j}=\frac{1}{j_{\lim}}+\frac{1}{j_{k}}\Rightarrow \;j_{k}=\frac{j\times j_{\lim}}{j_{\lim}-j}$$

*j*: current density of the disk (geometric). *j*_lim_: diffusion-limiting current density of the plateau (geometric), O_2_ mass transport limitation in electrolyte. *n_ex_*: overall passed number of electrons per molecule of O_2_. *F*: Faraday constant (*F* = 96 485 C mol^–1^). *D*: diffusion coefficient of O_2_ in the electrolyte (*D* = 1.8×10^–5^ cm^2^ s^–1^ in 1 M KOH). Ω: speed of RRDE (rpm). *C*: O_2_ concentration in the electrolyte (*C* = 7.8×10^–4^ mol cm^–3^ in 1 M KOH). ν: kinematic viscosity of the electrolyte (ν = 1.0×10^–2^ cm^2^ s^–1^ in 1 M KOH). *j_k_*: mass-corrected kinetic current density (absolute activity). If the disk current is I_D_(A), then *C* (mol cm^–3^); if the disk current is I_D_ (mA), then *C* (mol dm^–3^ ≡ mol L^–1^).

In 1 M KOH at 1600 rpm, the calculated diffusion-limiting current density of ORR is 3.6 and 1.8 mA cm^−2^ for *n_ex_* = 4 (complete reduction) and *n_ex_* = 2 (expected partial reduction), respectively. Figure 1e shows that the current density at low potentials is especially small for the material PANI-Co, which suggests a reduced electrocatalytic activity in driving high current density even though this material displays the highest electrochemically active surface area (Figure 1c). From Figure 1f, the trend in hydrogen peroxide selectivity is PANI-Co < PANI < PANI-Ni. Specifically, at 0.6 V_RHE_, HO_2_^−^(%) is 40, 75 and 65 for PANI, PANI-Ni and PANI-Co, respectively. Taken together, the results indicate that the augmentation of the number of the available electrochemical active sites does not directly translate into a high performance in hydrogen peroxide production. It can be assumed that the presence of nickel cations significantly increases the selectivity in hydrogen peroxide production. However, this remains a proof of concept without real applications, since such an electrocatalyst cannot be stable in aqueous media, as the metal cation would leave in the electrolyte during long-term application. Therefore, a stabilization step by thermal treatment is necessary to obtain a stable electrocatalyst. For this reason, we investigated the same three materials having undergone a first heat treatment under air—in this case study, the temperature of 350 °C was considered (on the basis of our previous study [20]).

Figure 2a–d show the obtained results for the three materials after the polymerization (5 °C, 13 h) and the stabilization (air, 350 °C, 2 h), PANI-TS, PANI-Ni-TS and PANI-Co-TS. Extended data are reported in Figure S4 for the double-layer capacitance measurements for determining the electrochemically active surface area (ECSA). Figure 2a shows a change in the CV profiles for PANI-Co, possibly because of the oxidation that suppresses the redox processes associated with the oxy–hydroxide transitions of M(OH)_2_ + OH^−^ = MOOH + H_2_O + e^−^ (M = Ni, Co), still present for the material PANI-Ni when considering the intensity of the peak at 1.25 V_RHE_ [40]. Figure 2d shows that the capacitance increases after this thermal treatment where the ECSA trend is PANI-NI (C_dl_ = 77 ± 3 µF) < PANI (C_dl_ = 92 ± 2 µF) < PANI-Co (C_dl_ = 104 ± 3 µF). For ORR, the difference in the LSV at 1600 rpm in O_2_-saturated 1 M KOH electrolyte (Figure 2c) is less important compared to the previous situation (Figure 1e). The hydrogen peroxide amount is nearly the same for PANI-Co, 0.6 V_RHE_, HO_2_^−^(%) ~40. For PANI-TS and PANI-Ni-TS, there is an inversion of the trend with HO_2_^−^(%) of ~77 and 65 at 0.6 V_RHE_, respectively. According to our previous XPS analysis, at this stage, the introduced metallic species are at their oxidized state [20]. Hence, it is interesting the examine whether the formation of real metallic species could change the trends in selectivity during ORR. Indeed, we recently showed that using thermal treatment under an inert atmosphere to generate a reductive environment will lead to the formation of mixture of metals and chalcogenides of NiS_x_ (x = 0, 2/3, 8/9 and 4/3) or CoS_x_ (x = 0 and 8/9) onto a carbon-nitrogen-sulfur nanostructured network [41].

**Figure 3:**
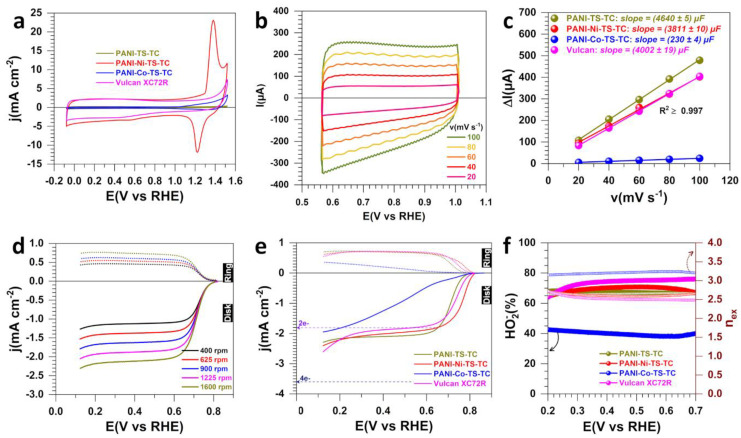
Electrochemical characterization for the materials obtained after polymerization (5 °C, 13 h), stabilization (air, 350 °C, 2 h) and calcination (N_2_, 900 °C, 6 h). (**a**) iR-drop uncorrected CV (N_2_-saturated 1 M KOH, 25 °C, 100 mV s^−1^, 0 rpm). (**b**) iR-drop uncorrected CV of PANI-TS-TC (N_2_-saturated 1 M KOH, 25 °C, 0 rpm) for determining ECSA. (**c**) Capacitive current (ΔI_a_ = I_a_−I_c_) vs. scan rate at E(V_RHE_) = 0.8 (PANI-TS-TC), 0.9 (PANI-Ni-TS-TC), 0.7 (PANI-Co-TS-TC) and 1.0 (Vulcan). (**d**) iR-drop uncorrected LSV of ORR for PANI-TS-TC at different speeds of RRDE (O_2_-saturated 1 M KOH, 25 °C, 5 mV s^−1^, E_ring_ = 1.2 V_RHE_). (**e**) iR-drop uncorrected LSV of ORR at RRDE (O_2_-saturated 1 M KOH, 25 °C, 5 mV s^−1^, 1600 rpm, E_ring_ = 1.2 V_RHE_). (**f**) HO_2_^−^% (left) and number of transferred electrons per molecule of O_2_ (right *y*-axis) from panel (**e**).

We next interrogated the impact of the three steps of polymerization (5 °C, 13 h), stabilization (air, 350 °C, 2 h) and calcination (N_2_, 900 °C, 6 h) on the electrochemical properties of ORR in 1 M KOH. The three materials are PANI-TS-TC, PANI-Ni-TS-TC and PANI-Co-TS-TC. To provide a fair comparison, a commercial carbon black, Vulcan XC72R, known for its ability in hydrogen peroxide production was used as a benchmark system. Figure 3a–f display the obtained results, while extended data are reported in Figures S5–S8. The overlay voltammograms in Figure 3a confirm the presence of the redox processes associated with the oxy–hydroxide transitions of Ni(OH)_2_ + OH^−^ = NiOOH + H_2_O + e^−^. The determined double-layer capacitance, proportional to the electrochemically active surface area, is C_dl_ = 4640 ± 5, 3811 ± 10, 230 ± 4 and 4002 ± 19 µF for PANI-TS-TC, PANI-Ni-TS-TC, PANI-Co-TS-TC and Vulcan, respectively. Except for the case of PANI-Co-TS-TC, this calcination dramatically increases the electrochemically active surface area by more than 10 times. The LSV curves in Figure 3c for ORR at PANI-TS-TC (other materials are reported in Figures S7 and S8) show a well-defined diffusion-limiting current of 2.2 mA cm^−2^ at 1600 rpm, which falls between 3.6 mA cm^−2^ for n_ex_ = 4 (complete reduction) and 1.8 mA cm^−2^ for n_ex_ = 2 (expected partial reduction). Furthermore, the overlay current–potential curves of Figure 3e show that the onset potential is close to the theoretically expected 0.75 V_RHE_ for the two-electron transferred process. We note that the PANI-Co-TS-TC material presents a linear profile, suggesting that it would present a large ohmic drop resistance, which is not the case, as the found value of 6 Ω will not noticeably change the trend after applying the iR-drop correction, considering the current rage of milliamps.

Figure 3f shows the quantitative analysis from the recorded LSV curves to concurrently visualize the current coming from the oxygen reduction and the ring current following the oxidation any produced hydrogen peroxide. At PANI-Co-TS-TC, HO_2_^−^(%) of ~40 is in the same range as the data from previous sections. It is noteworthy that, in the potential widow of interest, 0.5–0.7 V_RHE_, the other two materials PANI-TS-TC and PANI-Ni-TS-TC yield high current density (Figure 3e) and better selectivity in hydrogen peroxide production (Figure 3f) by approaching the state-of-the-art material, Vulcan. We note that, being a process at the cathode of the electrolysis cell, the electrode potential of ORR must be as high as possible to require the minimum electrical energy, since the cell voltage is given by the relationship U_(cell)_ = E_(anode)_ − E_(cathode)_. Specifically, at 0.6 V_RHE_, the selectivity towards the two-electron reduction is HO_2_^−^(%) = 65, 70 and 75 for PANI-TS-TC, PANI-Ni-TS-TC and Vulcan, respectively. The main outcome of these three previous studies is that after polymerization, heat treatment under air and inert atmosphere, the latter step allows one to target materials with a higher performance in terms of current density and selectivity towards the oxygen-to-hydrogen peroxide electro-conversion. This is achieved with polyaniline materials modified or not by nickel species. It is well-known that the electron transfer on graphitic materials strongly depends on the physicochemical and electronic properties of the electrode materials; however, the high electrocatalytic activity relies on the surface chemistry [42,43]. On the basis of our previous Raman and FTIR spectroscopy analysis [19,20], the relationship between the electron transfer and the crystallinity or nature of graphite species is discussed. For PANI-Ni, the most relevant electrocatalyst for HO_2_^-^ production, the Raman spectra exhibit four bands. Two bands corresponding to G and D were found between 1589 and 1594 cm^−1^ and between 1311 and 1331 cm^−1^, respectively. The band G reflects the degree of graphitization and the D band is related to edge defects and disordered induce feature. The third and fourth bands that appeared at fixed frequency at 1480 and 1165 cm^−1^ are characteristic of amorphous carbon and the presence of nanocrystalline diamond. The obtained *A_D_/A_G_* ratio was 3.1 with an in-plane crystallite size (*L_a_*) of 14.8. After thermal stabilization and heat treatment steps, the G-band position shifts and the intensity ratio of A_D_/A_G_ decreases to 2.2, while the in-plane crystallite size increases to 20.9 nm, indicating the transformation of graphite to an ordered structure with nanocrystalline graphite formation [44]. The A_D_/A_G_ ratio decreasing could reflect the sp2 carbon fraction raise and thus may be beneficial for the O_2_ molecule adsorption. Such observation is in line with the XPS result that showed the increase in C=C compared to C-O and C=O and after calcination [20]. It is widely recognized that the enhancement of the electron transfer rate is related to the defect engineering and to the higher density of step edges on the electrode surface as well as graphite distortion [42,45]. Earlier studies have demonstrated that the presence of quinone groups on the carbon surface, especially the semi-quinone radical anions (Q^•−^), play a crucial role for the enhancement of HO_2_^-^ production in an alkaline medium; the mechanism described by Equations (12)–(16) [46,47]. When O_2_ is adsorbed on the surface of the electrode, O_2_^•−^ is formed by Equation (13), which remains adsorbed on the electrode surface and can be protonated by H_2_O (Equation (14)). However, in alkaline media, HO_2_^•−^_(ads)_ possesses higher p*K*a than HO_2_^−^_(aq)_, which hindered its protonation, leading to the formation of hydrogen peroxide as a final product [48].

Q + e^−^ → Q^•−^ (12)

Q^•−^ + O_2_ → Q-O_2_^•−^_(ads)_ (13)

Either

Q-O_2_^•−^ + H_2_O → Q + HO_2_^•^_(ads)_ + OH^−^ (14)

HO_2_^•^_(ads)_ + e^−^ → HO_2_^−^_(aq)_ (15)

Or

Q-O_2_^•−^ + H_2_O + e^−^ → Q + HO_2_^−^ + OH^−^ (16)

where Q is the surface quinone species.

Based on this finding, we selected the most efficient materials, PANI-TS-TC and PANI-Ni-TS-TC, for further studies in order to get closer to the real conditions of use in electrolyzers. The state-of-the art material, Vulcan, was also used for comparison.

**Figure 4:**
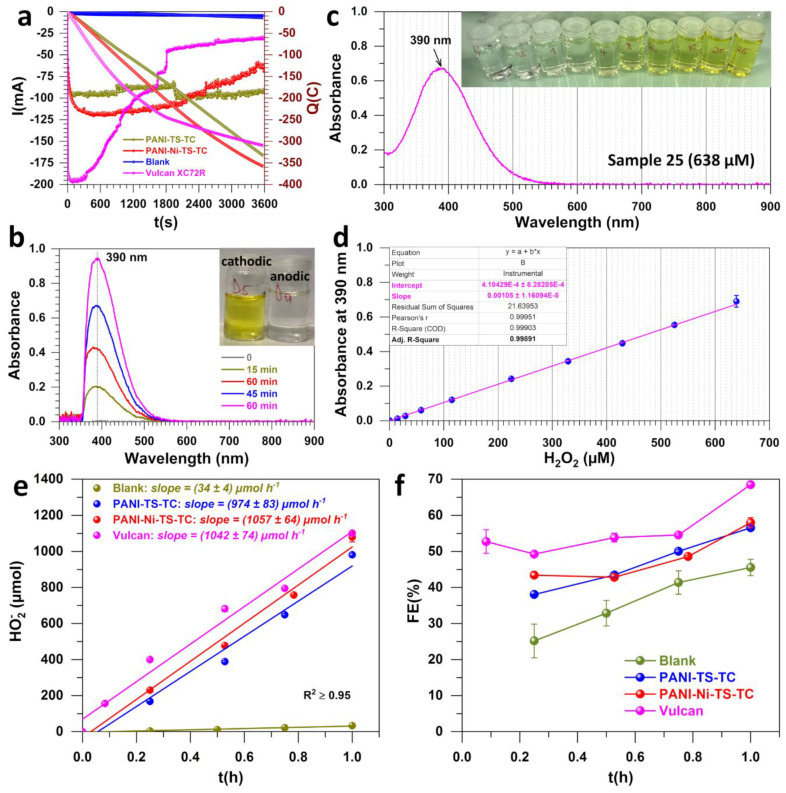
Bulk electrolysis and electroanalysis. (**a**) Electrical charge passed during the potentiostatic electrolysis at an applied potential of 0.6 V_RHE_ (iR-uncorrected). (**b**) UV-vis spectra of the anodic compartment at different times of the electrolysis (aliquot was neutralized by sulfuric acid before addition of potassium titanium oxalate solution). (**c**) UV-vis spectrum of hydrogen peroxide (638 µM) in the presence of sulfuric and potassium titanium oxalate solution: inset shows the color of the solution in the presence of increasing concentration of hydrogen peroxide. (**d**) Calibration curves from UV-vis assays at 390 nm and from solutions of panel (**c**). (**e**) Hydrogen peroxide productivity as quantified by the UV-vis assays. (**f**) Faradaic efficiency. The electrocatalyst loading was 0.5 mg cm^−2^ and the electrode size was 4 cm^2^. The blank electrode was bare carbon paper MGL370 (Fuel Cell Earth LLC). Error bars represent 1 SD (n ≥ 3).

Therefore, to confirm the observed efficiency with the RRDE method, hydrogen peroxide was monitored spectrophotometrically after bulk electrolysis, which was performed by loading the electrocatalysts onto gas diffusion electrodes (3D network of carbon microfibers, carbon paper MGL370, Fuel Cell Earth LLC) and testing them in a H-type cell made of two compartments separated by a hydroxide anion exchange membrane. Figure 4a shows the obtained potentiostatic curves of current vs. time at an applied potential of 0.6 V_RHE_ where the electrocatalyst loading was 0.5 mg cm^−2^ and the electrode size was 4 cm^2^. The control experiment was performed using the blank material (no deposit of the catalyst ink), which does not show any significant current. The electrocatalyst PANI-TS-TC maintains a stable current of an absolute value of 100 mA, while the major loss of the current is observed for Vulcan. The decrease in the current after a certain duration for electrocatalysts delivering high currents could be due to the mass transport issues in the vicinity of the electrode surface, particularly for such 3D supports. To find the best condition of UV-vis titration, we recorded U-Vis spectra in full range of 300–900 nm. Figure 4b shows the UV-vis spectra for solutions obtained at different times of electrolysis for the material PANI-Ni-TS-TC and after neutralization by sulfuric acid plus potassium titanium oxalate to complex with hydrogen peroxide. Meanwhile, standard solutions were prepared and tested, as reported in Figure 4c. Figure 4d highlights that there is a linear correlation at the maximum wavelength of λ = 390 nm. The series of control experiments confirm that this wavelength from the pertitanic acid complex between potassium titanium oxalate and hydrogen peroxide can be used to selectively quantify any hydrogen peroxide, similar to previous reports [26,27,28,29]. The productivity of hydrogen peroxide as quantified by the UV-vis assays is displayed in Figure 4c. The faradaic efficiency, as the ratio between the electrical charge corresponding the quantified hydrogen peroxide and the total charge (from Figure 4a), is shown in Figure 4f. Comparative data with the literature are reported in Table S1. Specifically, the production rate is 34 ± 4, 974 ± 83, 1057 ± 64 and 1042 ± 74 µmol_H2O2_ h^−1^ for Blank, PANI-TS-TC, PANI-Ni-TS-TC, and Vulcan, respectively. Otherwise, it is 521 ± 37, 487 ± 42 and 529 ± 32 mol_H2O2_ kg^−1^_cat_ h^−1^ for Vulcan, PANI-TS-TC and PANI-Ni-TS-TC, respectively. Taking into account the electrode area, the production rate is 130 ± 9, 122 ± 10 and 132 ± 8 mol_H2O2_ kg^−1^_cat_ cm^−2^ for the same order. The faradaic efficiency of PANI-TS-TC and PANI-Ni-TS-TC is similar, FE = (58 ± 2)%, while that of Vulcan is FE = (68 ± 1)%. The discrepancy in terms of the faradaic efficiency between the RRDE method and the bulk electrolysis followed by UV-vis titration means that the electrocatalytic behavior using a thin-film method is not necessary to what is happening in real systems that use three-dimensional supports such as gas diffusion electrodes [49,50]. For comparison with the existing data in the literature (see Table S1 for all metrics), the faradaic efficiency is below the recorded values of 94–95% [8,51], but is comparable to up-to-date data gathered in Ref. [3], e.g., faradaic efficiency of 70–87% [52,53].

### 3.2. Structural Analysis

**Figure 5:**
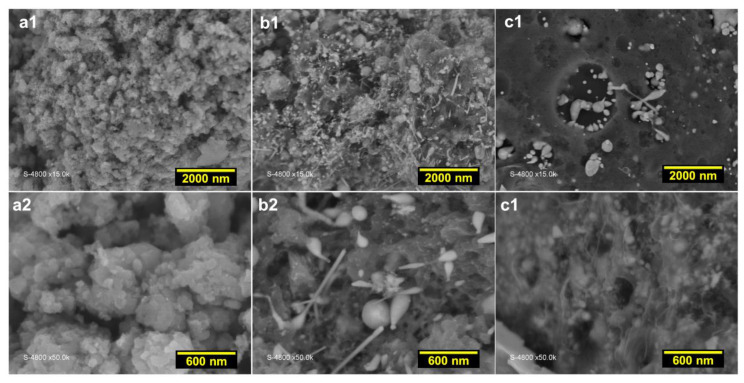
SEM characterization of the materials obtained after polymerization (5 °C, 13 h), stabilization (air, 350 °C, 2 h) and calcination (N_2_, 900 °C, 6 h): backscattered SEM images at different magnification for: (**a1**,**a2**) PANI-TS-TC, (**b1**,**b2**) PANI-Ni-TS-TC, and (**c1**,**c2**) PANI-Co-TS-TC.

The three obtained materials after polymerization (5 °C, 13 h), stabilization (air, 350 °C, 2 h) and calcination (N_2_, 900 °C, 6 h) were analyzed by SEM, EDX and XRD to obtain structure information. Other extensive characterization results can be found in Ref. [20]. To better visualize the formed structures, backscattered SEM images were recorded at different magnifications as displayed in Figure 5a1 to 5c2. The comparison between the control material PANI-TS-TC (Figure 5a1,a2) and those containing the metallic species PANI-Ni-TS-TC (Figure 5b1,b2) and PANI-Co-TS-TC (Figure 5c1,c2) shows the presence of micro-structured particles of different shape and size. For polyaniline-based materials, it is known that the thermal treatment under an inert atmosphere of argon or nitrogen at 600–1200 °C will trigger the rearrangement of the different atoms to create new bonds of carbon–carbon (C-C, C=C, C-H) and carbon–nitrogen (pyridinic-N, pyrrolic-N, and graphitic-N) in a nanostructured network that has different levels of electrical conductivity, number of active sites and electrocatalytic kinetics [18,20,54,55,56,57,58]. In order to obtain a qualitative analysis of the nature of the structures formed with metallic species, in-depth analysis was conducted by EDX.

**Figure 6:**
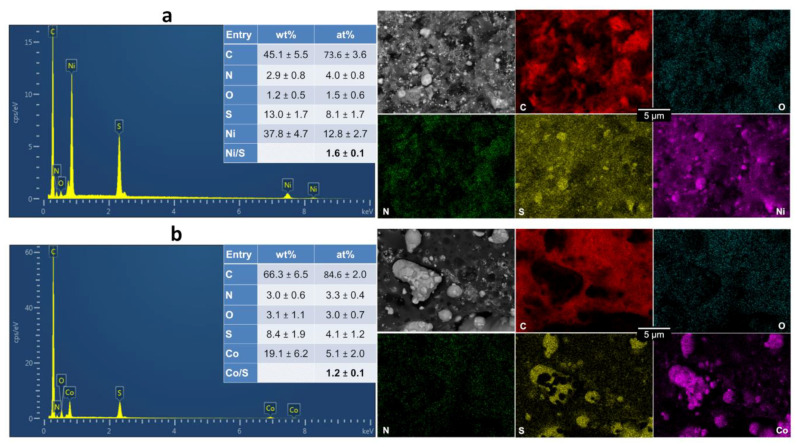
Materials obtained after polymerization (5 °C, 13 h), stabilization (air, 350 °C, 2 h) and calcination (N_2_, 900 °C, 6 h): EDX spectra, backscattered SEM images plus EDX maps of: (**a**) PANI-Ni-TS-TC, and (**b**) PANI-Co-TS-TC.

Figure 6a,b show the obtained EDX spectra, backscattered SEM images and the mapping. A closer observation conforms the formation of nickel-sulphur or cobalt-sulphur species and that there is no other overlapping between the signals of the metals and oxygen, carbon or nitrogen. For PANI-Ni-TS-TC, the atomic ratio of Ni/S is 1.6 ± 0.1, which suggests the formation of a Ni_3_S_2_ phase, while for PANI-Co-TS-TC, the atomic ratio of 1.2 ± 0.1 is in line with the formation of Co_9_S_8_ species [41]. It was previously observed that the length of the tip of the Ni_3_S_2_ particles increases with the duration of the calcination step [20], explained by the so-called vapor-liquid-solid (VLS) mechanism of growth, which involves a vapor of nickel-sulfur, meaning that the super-saturation and nucleation at the liquid/solid interface leads to axial crystal growth [59].

**Figure 7:**
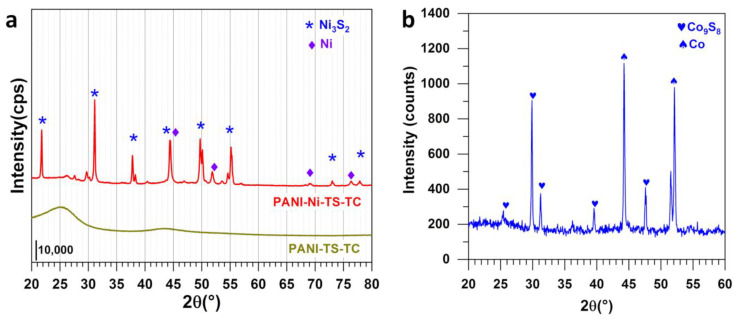
XRD patterns the electrocatalysts obtained after polymerization (5 °C, 13 h), stabilization (air, 350 °C, 2 h) and calcination (N_2_, 900 °C, 6 h): (**a**) PANI-TS-TC and PANI-Ni-TS-TC, and (**b**) PANI-Co-TS-TC.

XRD results are reported in Figure 7a,b. For PANI-Ni-TS-TC, the suggested structure of Ni_3_S_2_ from EDX analysis is confirmed by its main diffraction peaks at 2θ = 22°, 31°, 38°, 44°, 50.0° and 55 for (101), (110), (003)/(021), (202), (113)/(211) and (122), respectively (JCPDS n° 44-1418) [60,61,62,63,64,65]. This structure co-exists with Ni (JCPDS 03-1051). For PANI-Co-TS-TC, Co_9_S_8_ (JCPDS 86-2273) co-exists with Co (JCPDS 15-0806). It can be concluded that the electrocatalysts are heterogeneous. It will be interesting in future studies to obtain pure phases of each of these compounds and to assess how the efficiencies in ORR in hydrogen peroxide production change.

## 4. Conclusions

In this study, we investigated the applicability of materials derived from the conducting polymer polyaniline (PANI) for oxygen-to-hydrogen peroxide electro-conversion in an alkaline medium. Indeed, a recent procedure (*Front. Chem.* *2020*, *8*, *Article number 385*) highlighted the formation of self-supported electrocatalytic materials by the oxidative polymerization of aniline into polyaniline with notable performance when tested for the reduction of H_2_O to H_2_ in alkaline media. Herein, at each stage of the synthesis (polymerization (5 °C, 13 h), stabilization (air, 350 °C, 2 h) and calcination (N_2_, 900 °C, 6 h)), three materials of PANI, modified or not by nickel or cobalt species, were used to study the oxygen reduction reaction (ORR) and probe the selective two electrons pathway (O_2_ + H_2_O + 2e^−^ → HO_2_^−^ + HO^−^; HO_2_^−^ is a hydroperoxide anion, the most stable specie in alkaline electrolytes, p*K*a(H_2_O_2_/HO_2_^−^) = 11.75). We employed two complementary electroanalytical step-ups, namely a rotating ring-disk electrode (RRDE) and bulk electrolysis combined with UV-vis titration. Using the RRDE method, the selectivity towards hydrogen peroxide production was higher than 60% for polyaniline and nickel-modified polyaniline. The third step, the calcination (N_2_, 900 °C, 6 h), produced the highest current density and selectivity of 65–80%, which drops to 40% in the presence of cobalt where XRD analysis revealed the co-existence of Co_9_S_8_ with Co, while Ni_3_S_2_ and Ni were the main species when the polymerization was performed in the presence of nickel salt. From the bulk electrolysis and the spectrophotometry, the production rate was 974 ± 83, 1057 ± 64 and 1042 ± 74 µmol_H2O2_ h^−1^ for calcined polyaniline, calcined nickel-modified polyaniline and Vulcan XC72R (state-of-the-art electrocatalyst), respectively. These results correspond to 521 ± 37, 487 ± 42 and 529 ± 32 mol_H2O2_ kg^−1^_cat_ h^−1^ (130 ± 9, 122 ± 10 and 132 ± 8 mol_H2O2_ kg^−1^_cat_ cm^−2^) for Vulcan, calcined polyaniline and calcined nickel-modified polyaniline, respectively. The faradaic efficiency was 58–78%. The structural analysis showed that the electrocatalysts are heterogeneous, so it would be worthwhile in future studies to target pure phases of each of the preceding compounds and to evaluate the influence on the efficiencies in ORR in hydrogen peroxide production. The present work contributes towards the engineering of efficient electrocatalysts derived from polymers for the decentralized electrosynthesis of hydrogen peroxide, which is of key importance in chemical industries.

## Data Availability

The data supporting the findings of this study are available within the paper. Any other relevant data are also available upon reasonable request from the corresponding author.

## Supplementary Materials

The following supporting information can be downloaded at: www.mdpi.com/article/10.3390/polym14030607/s1, Figures S1–S8: Extended electrochemical characterization data; Table S1: Summary of reported electrocatalysts for hydrogen peroxide production in aqueous electrolyte.
