# Supplementary material for: Probing Oxygen-to-Hydrogen Peroxide Electro-Conversion at Electrocatalysts Derived from Polyaniline
Source: Polymers (Basel). 2022 Feb 4;14(3):607. doi: 10.3390/polym14030607 (PMC8839311; doi:10.3390/polym14030607)
Supplement: Supplementary file 1 [file polymers-14-00607-s001.zip › polymers-1560772-supplementary.pdf]

Supporting Information (SI) for

Probing Oxygen-to-Hydrogen Peroxide Electro-conversion at Electrocatalysts Derived from Polyaniline

Yaovi Holade[1,*] Sara Knani[1], Marie-Agnès Lacour[2], Julien Cambedouzou[1], Sophie Tingry[1], Teko W. Napporn[3] and David Cornu[1]

[1] Institut Européen des Membranes, IEM UMR 5635, Univ Montpellier, ENSCM, CNRS, Montpellier, France; sarra.knani@umontpellier.fr (S.K.), julien.cambedouzou@enscm.fr (J.C.), david.cornu@enscm.fr (D.C.), sophie.tingry@umontpellier.fr (S.T.)

[2] ChemLab, ChemLab, ENSCM, 34296 Montpellier, France; marie-agnes.lacour@enscm.fr (M.-A.L.)

[3] Université de Poitiers, IC2MP UMR-CNRS 7285, 86073 Poitiers Cedex 9, France; teko.napporn@univ-poitiers.fr (T.W.N)

* Correspondence: yaovi.holade@enscm.fr ; Tel.: +33-467-14-92-94

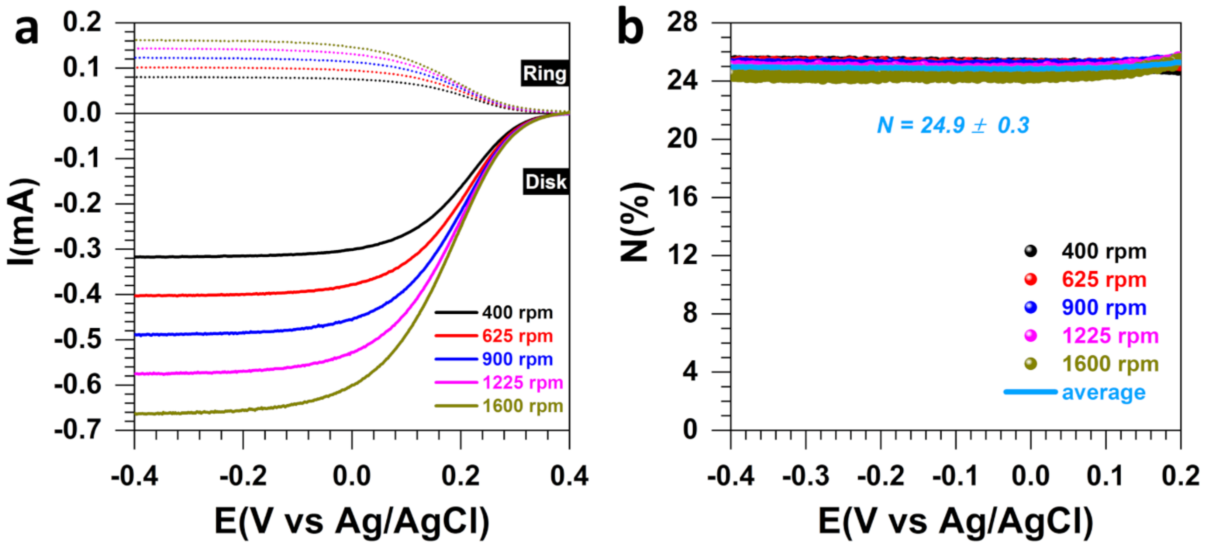

Figure S1. Determination of the collection efficiency of the RRDE setup. **(a)** LSV recorded at different speeds in N_2-saturated 1 M KNO_3 electrolyte in the presence of 5 mM $K_3[Fe(CN)_6]$ at 5 mV s^{-1} and room temperature (the ring potential was 0.5 V vs Ag/AgCl). **(b)** Corresponding collection efficiency (N), average = 24.9 ± 0.3%, in agreement with the manufacturer's value of 24.9%.

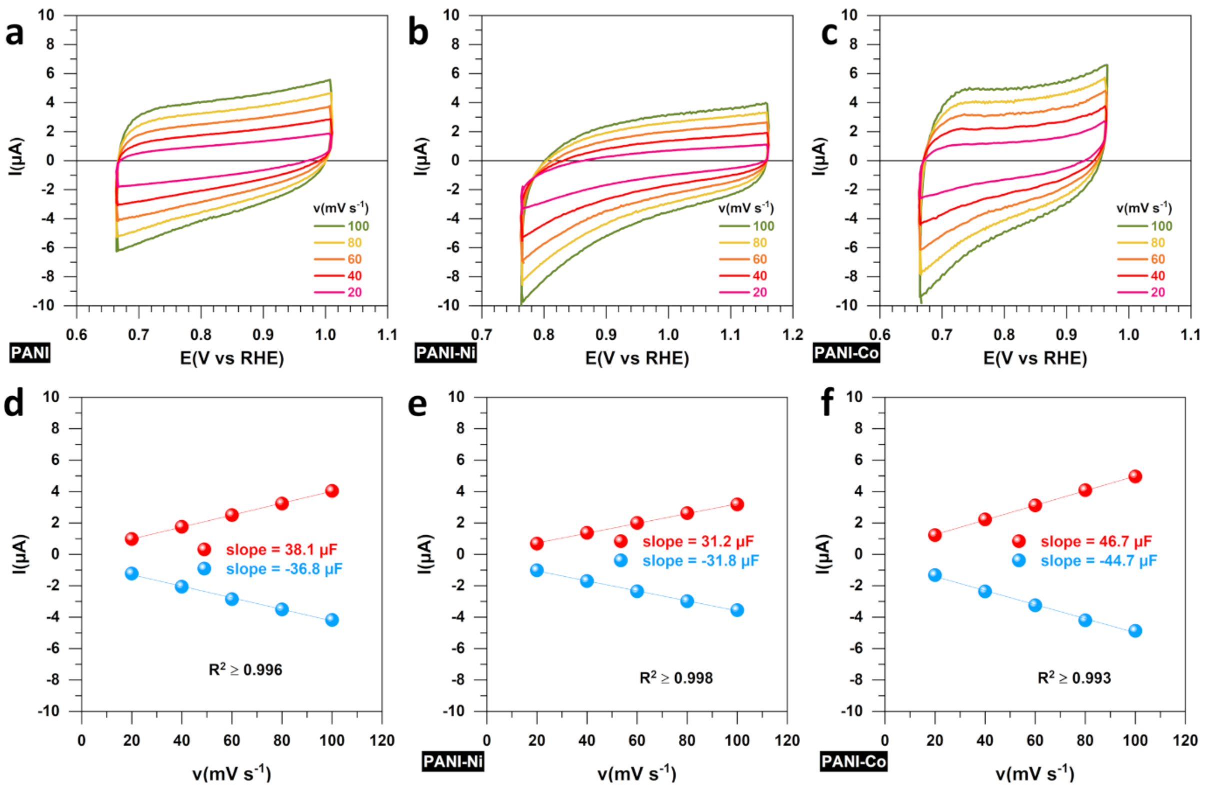

Figure S2. Electrochemical characterization for the materials obtained after polymerization (5 °C, 13 h). Double-layer capacitance measurements for determining electrochemically active surface area (ECSA): **(a-c)** iR-uncorrected CVs recorded at different scan rates in the double-layer capacitance region (N_2-saturated 1 M KOH, 25 °C, 0 rpm) and **(d-f)** The anodic (I_a) and cathodic (I_c) charging currents measured at $E(V_{RHE})$ = 0.8 (PANI), 1.0 (PANI-Ni) and 0.8 (PANI-Co) and plotted as a function of scan rate. **(a,d)** PANI, **(b,e)** PANI-Ni and **(c,f)** PANI-Co.

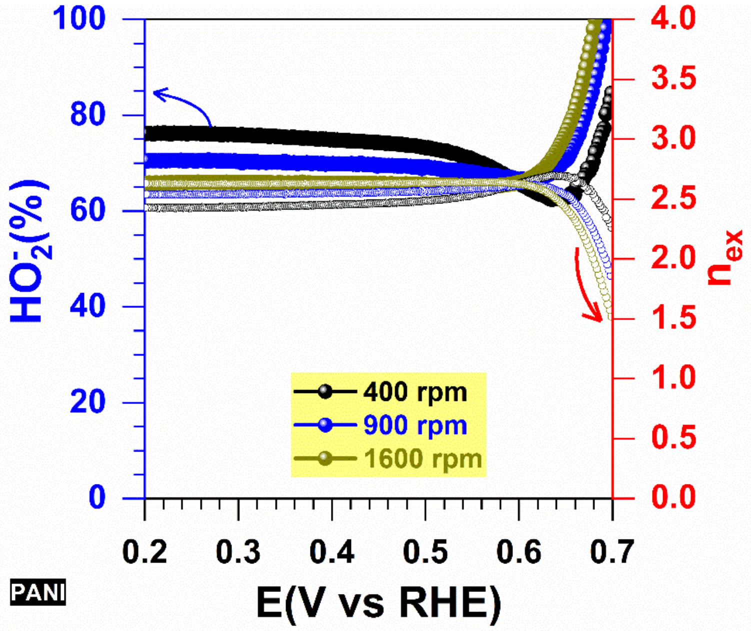

Figure S3. HO_2^- % (left) and number of transferred electrons per molecule of O_2 (right y-axis) from iR-drop uncorrected LSV of ORR for PANI at different speeds of RRDE (O_2-saturated 1 M KOH, 25 °C, 5 mV s^{-1}, E_{ring} = 1.2 V_{RHE}).

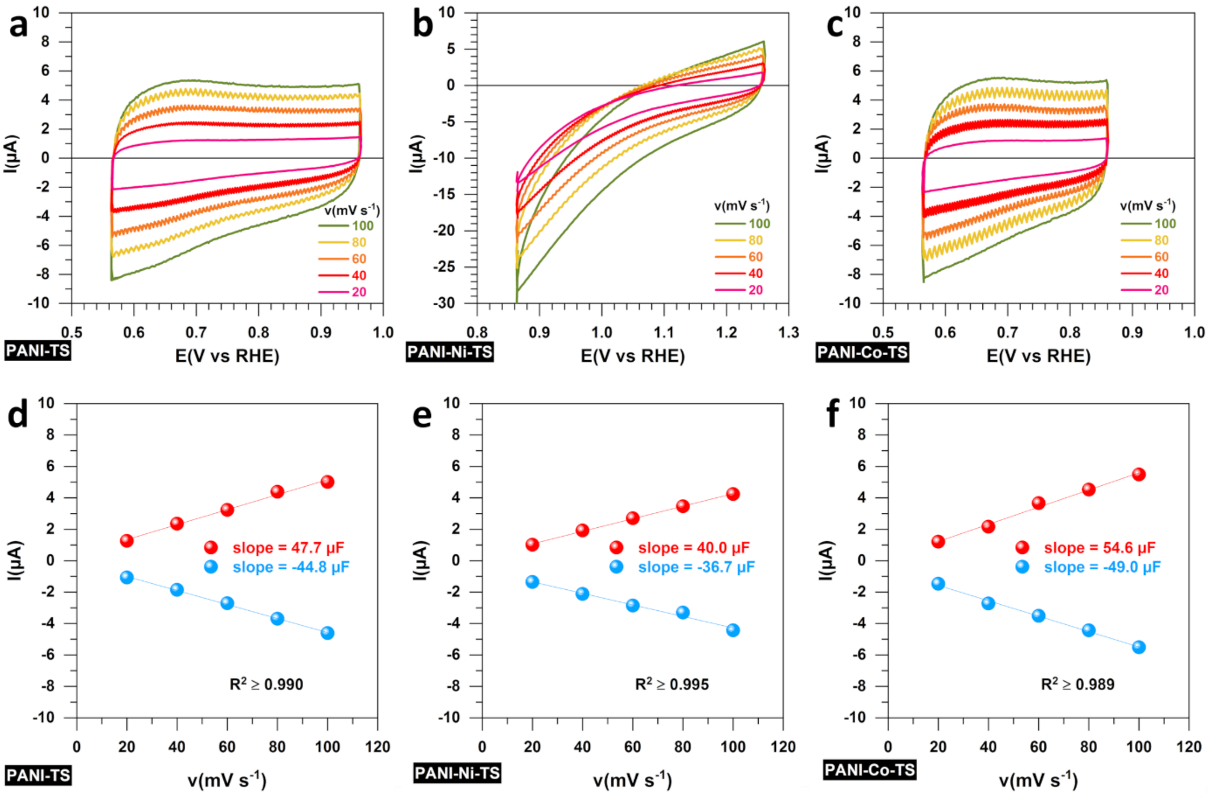

Figure S4. Electrochemical characterization for the materials obtained after polymerization (5 °C, 13 h) and stabilization (air, 350 °C, 2 h). Double-layer capacitance measurements for determining electrochemically active surface area (ECSA): **(a-c)** iR-uncorrected CVs recorded at different scan rates in the double-layer capacitance region (N_2-saturated 1 M KOH, 25 °C, 0 rpm) and **(d-f)** The anodic (I_a) and cathodic (I_c) charging currents measured at $E(V_{RHE})$ = 0.8 (PANI-TS), 1.2 (PANI-Ni-TS) and 0.7 (PANI-Co-TS) and plotted as a function of scan rate. **(a,d)** PANI-TS, **(b,e)** PANI-Ni-TS and **(c,f)** PANI-Co-TS.

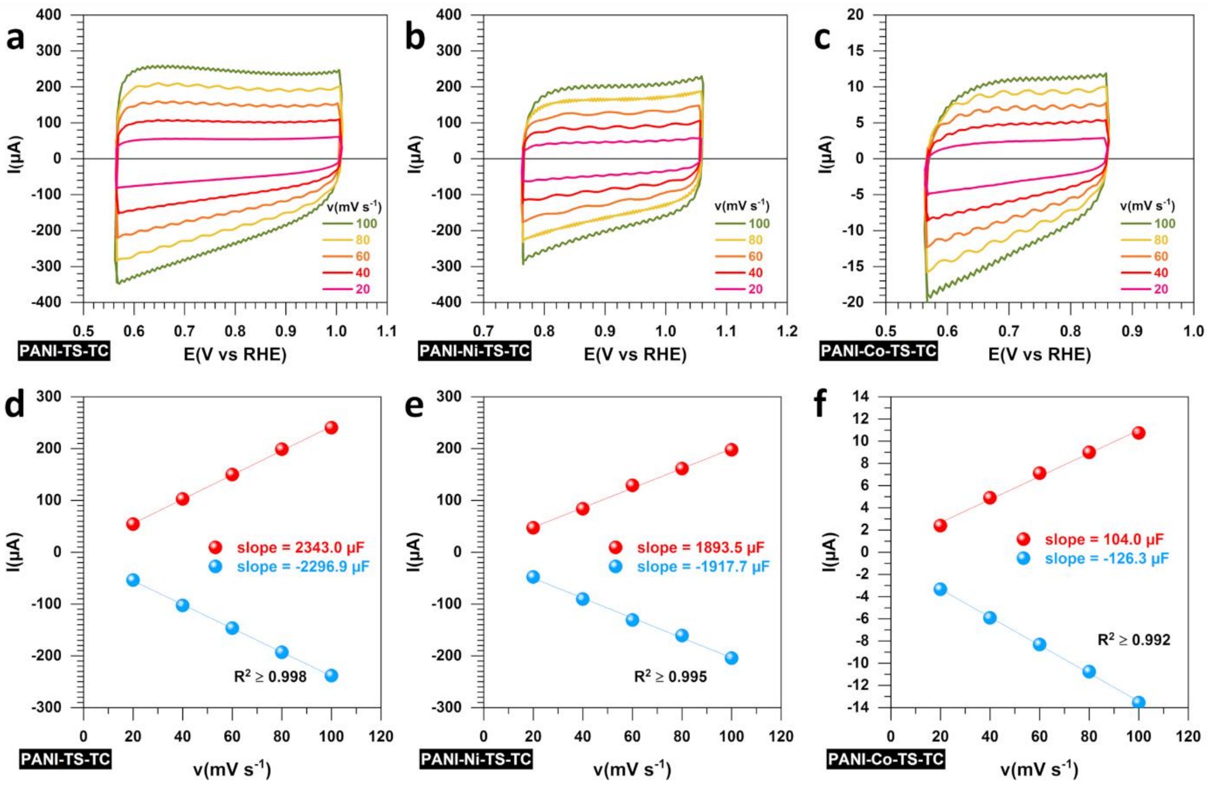

Figure S5. Electrochemical characterization for the materials obtained after polymerization (5 °C, 13 h), stabilization (air, 350 °C, 2 h) and calcination (N_2, 900 °C, 6 h). Double-layer capacitance measurements for determining electrochemically active surface area (ECSA): **(a-c)** iR-uncorrected CVs recorded at different scan rates in the double-layer capacitance region (N_2-saturated 1 M KOH, 25 °C, 0 rpm) and **(d-f)** The anodic (I_a) and cathodic (I_c) charging currents measured at $E(V_{RHE})$ = 0.8 (PANI-TS-TC), 0.9 (PANI-Ni-TS-TC) and 0.7 (PANI-Co-TS-TC) and plotted as a function of scan rate. **(a,d)** PANI-TS-TC, **(b,e)** PANI-Ni-TS-TC and **(c,f)** PANI-Co-TS-TC.

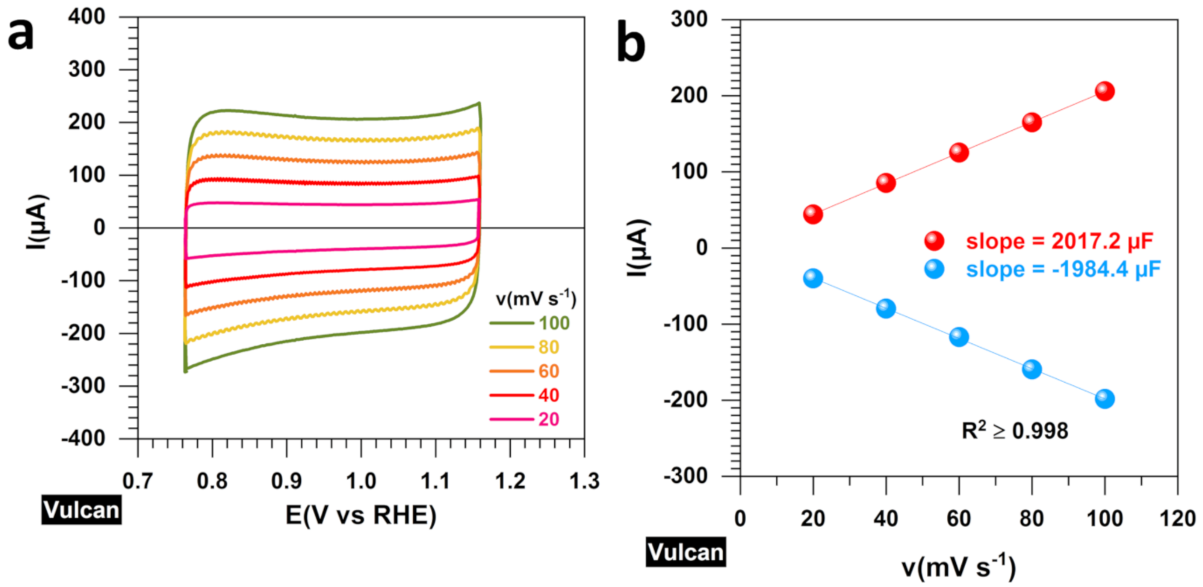

Figure S6. Electrochemical characterization of commercial carbon black, Vulcan XC72R. Double-layer capacitance measurements for determining electrochemically active surface area (ECSA): **(a)** iR-uncorrected CVs recorded at different scan rates in the double-layer capacitance region (N_2-saturated 1 M KOH, 25 °C, 0 rpm) and **(b)** The anodic (I_a) and cathodic (I_c) charging currents measured at $E(V_{RHE}) = 1.0$ and plotted as a function of scan rate.

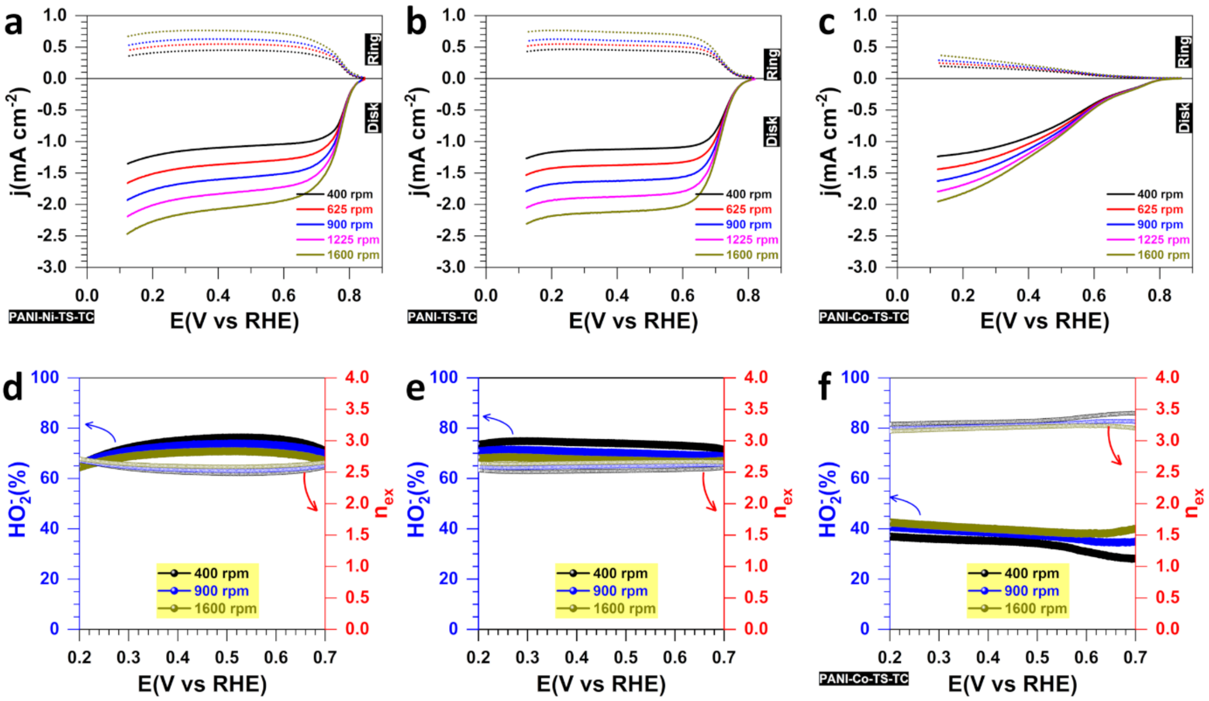

Figure S7. Electrocatalytic properties of the materials obtained after polymerization (5 °C, 13 h), stabilization (air, 350 °C, 2 h) and calcination (N_2, 900 °C, 6 h). **(a-c)** iR-drop uncorrected LSV of ORR at different speeds of RRDE (O_2-saturated 1 M KOH, 25 °C, 5 mV s^{-1}, E_{ring} = 1.2 V_{RHE}). **(d-f)** HO_2^- % (left) and number of transferred electrons per molecule of O_2 (right y-axis) from panels **(a-c)**. **(a,d)** PANI-Ni-TS-TC, **(b,e)** PANI-TS-TC and **(c,f)** PANI-Co-TS-TC.

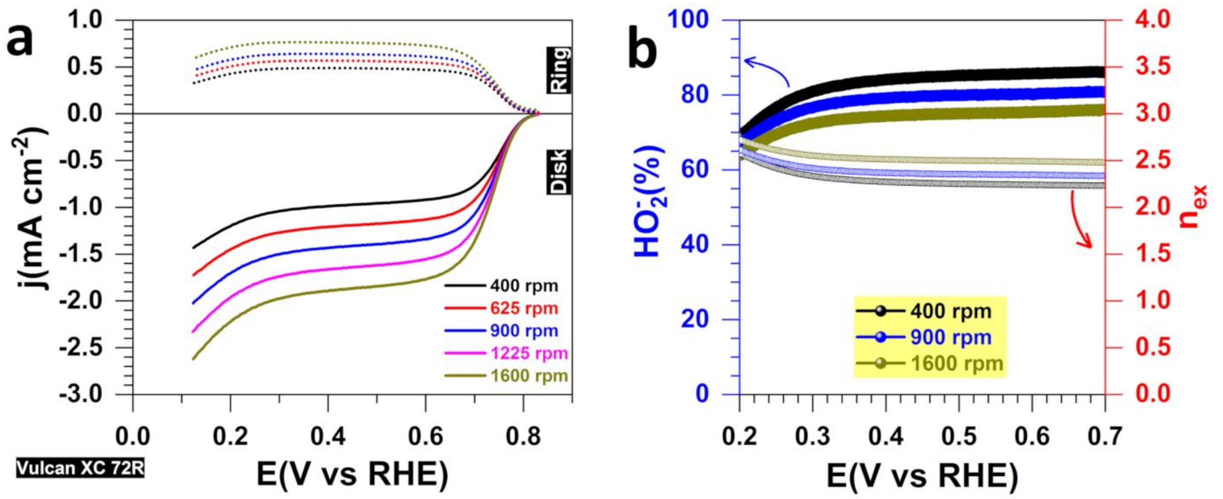

Figure S8. Electrocatalytic properties of commercial carbon black, Vulcan XC72R. **(a)** iR-drop uncorrected LSV of ORR at different speeds of RRDE (O_2-saturated 1 M KOH, 25 °C, 5 mV s^{-1}, E_{ring} = 1.2 V_{RHE}). **(b)** HO_2^- % (left) and number of transferred electrons per molecule of O_2 (right y-axis) from panels **(a)**.

Table S1. Summary of reported electrocatalysts for hydrogen peroxide production in aqueous electrolyte. FE = faradaic efficiency. n.d. = note determined.

Ref	Experimental Conditions	Electroanalytical by RRDE			Productivity by Analytical methods after electrolysis		
		Selectivity (%)	n_{ex}	j_k(A g^{-1})	mol kg^{-1}_{cat} cm^{-2}	mol kg^{-1}_{cat} h^{-1}	FE (%)
Here	Vulcan XC72R in 1 M KOH	75	2.5	n.d.	130 ± 9	521 ± 37	68.5 ± 0.8
	PANI-TS-TC in 1 M KOH	65	2.6	n.d.	122 ± 10	487 ± 42	58.0 ± 1.4
	PANI-Ni-TS-TC in 1 M KOH	70	2.6	n.d.	132 ± 8	529 ± 32	56.6 ± 0.5
J. Mater. Chem. A 2020, 8 (36), 18840-18855.[1]	Bismuth-based materials (sample R30) in 1 M KOH	96	2.1	30 (at 0.6 V vs RHE) 930 (at 0.55 V vs RHE)	69	138	92
Chang, Q. et al.[2] Nat. Commun. 2020, 11, 2178.	$Pd^{\delta+}$-OCNT in 0.1 M $HClO_4$	95	n.d.	597 (at 0.55 V vs RHE)		1701	87
Xia, C. et al.[3] Science 2019, 366, 226-231.	Carbon black in 1 M Na_2SO_4 (pH7)	98	n.d.	n.d.	n.d.	3660	90-95
San Roman, D. et al.[4] ACS Catal. 2020, 10, 1993-2008.	nanowire-templated out-of-plane three-dimensional fuzzy graphene (NT-3DFG) in 0.1 M KOH	n.d.	2.5-3	n.d.	n.d.	n.d.	94
Ledendecker, M. et al.[5] ACS Catal. 2020, 5928-5938.	PdClx/C in 0.1 $HClO_4$	90	n.d.	73 (potential not specified)	n.d.	n.d.	n.d.
Sun, Y. et al. [6] ACS Catal. 2018, 8, 2844-2856.	Nitrogen-Doped Mesoporous Carbon Catalysts in 0.1 M KOH	85	2.1	n.d.	n.d.	562	70
Zakaria, M. B. et al.[7] J. Mater. Chem. A 2016, 4, 9266-9274.	Mn-Ru oxide in 0.1 M NaOH	100	2.0	n.d.	n.d.	n.d.	n.d.
Kim, H. W. et al.[8] Nature Catalysis 2018, 1, 282-290.	mild reduction of graphene oxide (mrGO) in 0.1 M KOH	100	2.0	110 (at 0.6 V vs RHE)	n.d.	n.d.	n.d.
ACS Appl. Mater. Interfaces. 2021, 13 (1), 382-390.[9]	$Sr_{0.7}Na_{0.3}Si_{0.95}Ni_{0.05}O_{3-\delta}$ in 0.1 M KOH	83	2.4-2.9	n.d	n.d	n.d	65
J. Am. Chem. Soc. 2019, 141 (31), 12372-12381.[10]	Co-N-C in 0.1 M KOH	65	2.7	n.d	n.d	4330	80

J. Colloid Int. Sci. 2021, 602, 799-809.[11]	B/N-C-2 in 0.1 M KOH	94	2	n.d	n.d	n.d	n.d
Nanoscale Horiz. 2020, 5 (5), 832-838.[12]	MnO/N@NC in 0.1 M $HClO_4$	80	2	n.d	n.d	n.d	n.d
Note for up-to-date data in May 2020: Readers are suggested to refer to Table 1 of a recent Perspective:[13] Jung, E.; Shin, H.; Hooch Antink, W.; Sung, Y.-E.; Hyeon, T., "Recent Advances in Electrochemical Oxygen Reduction to H_2O_2: Catalyst and Cell Design. ACS Energy Lett. 2020, DOI: 10.1021/acsenergylett.0c00812, 1881-1892". Publication Date: May 8, 2020. https://doi.org/10.1021/acsenergylett.0c00812.							

References

1. Morandi, P.; Flaud, V.; Tingry, S.; Cornu, D.; Holade, Y., Tartaric acid regulated the advanced synthesis of bismuth-based materials with tunable performance towards the electrocatalytic production of hydrogen peroxide. *Journal of Materials Chemistry A* **2020,** *8* (36), 18840-18855. http://dx.doi.org/10.1039/D0TA06466A.
2. Chang, Q.; Zhang, P.; Mostaghimi, A. H. B.; Zhao, X.; Denny, S. R.; Lee, J. H.; Gao, H.; Zhang, Y.; Xin, H. L.; Siahrostami, S.; Chen, J. G.; Chen, Z., Promoting H_2O_2 production via 2-electron oxygen reduction by coordinating partially oxidized Pd with defect carbon. *Nature Communications* **2020,** *11* (1), 2178. https://doi.org/10.1038/s41467-020-15843-3.
3. Xia, C.; Xia, Y.; Zhu, P.; Fan, L.; Wang, H., Direct electrosynthesis of pure aqueous H_2O_2 solutions up to 20% by weight using a solid electrolyte. *Science* **2019,** *366* (6462), 226-231. https://doi.org/10.1126/science.aay1844.
4. San Roman, D.; Krishnamurthy, D.; Garg, R.; Hafiz, H.; Lamparski, M.; Nuhfer, N. T.; Meunier, V.; Viswanathan, V.; Cohen-Karni, T., Engineering Three-Dimensional (3D) Out-of-Plane Graphene Edge Sites for Highly Selective Two-Electron Oxygen Reduction Electrocatalysis. *ACS Catalysis* **2020,** *10* (3), 1993-2008. https://doi.org/10.1021/acscatal.9b03919.
5. Ledendecker, M.; Pizzutilo, E.; Malta, G.; Fortunato, G. V.; Mayrhofer, K. J. J.; Hutchings, G. J.; Freakley, S. J., Isolated Pd Sites as Selective Catalysts for Electrochemical and Direct Hydrogen Peroxide Synthesis. *ACS Catalysis* **2020,** *10* (10), 5928-5938. https://doi.org/10.1021/acscatal.0c01305.
6. Sun, Y.; Sinev, I.; Ju, W.; Bergmann, A.; Dresp, S.; Kühl, S.; Spöri, C.; Schmies, H.; Wang, H.; Bernsmeier, D.; Paul, B.; Schmack, R.; Kraehnert, R.; Roldan Cuenya, B.; Strasser, P., Efficient Electrochemical Hydrogen Peroxide Production from Molecular Oxygen on Nitrogen-Doped Mesoporous Carbon Catalysts. *ACS Catalysis* **2018,** *8* (4), 2844-2856. https://doi.org/10.1021/acscatal.7b03464.
7. Zakaria, M. B.; Li, C.; Pramanik, M.; Tsujimoto, Y.; Hu, M.; Malgras, V.; Tominaka, S.; Yamauchi, Y., Nanoporous Mn-based electrocatalysts through thermal conversion of cyano-bridged coordination polymers toward ultra-high efficiency hydrogen peroxide production. *Journal of Materials Chemistry A* **2016,** *4* (23), 9266-9274. http://dx.doi.org/10.1039/C6TA01470D.
8. Kim, H. W.; Ross, M. B.; Kornienko, N.; Zhang, L.; Guo, J.; Yang, P.; McCloskey, B. D., Efficient hydrogen peroxide generation using reduced graphene oxide-based oxygen

reduction electrocatalysts. *Nature Catalysis* **2018,** *1* (4), 282-290. https://doi.org/10.1038/s41929-018-0044-2.

9. Thundiyil, S.; Kurungot, S.; Devi, R. N., Efficient Electrochemical Oxygen Reduction to Hydrogen Peroxide by Transition Metal-Doped Silicate Sr0.7Na0.3SiO3−δ. *ACS Applied Materials & Interfaces* **2021,** *13* (1), 382-390. https://doi.org/10.1021/acsami.0c16311.

10. Sun, Y.; Silvioli, L.; Sahraie, N. R.; Ju, W.; Li, J.; Zitolo, A.; Li, S.; Bagger, A.; Arnarson, L.; Wang, X.; Moeller, T.; Bernsmeier, D.; Rossmeisl, J.; Jaouen, F.; Strasser, P., Activity–Selectivity Trends in the Electrochemical Production of Hydrogen Peroxide over Single-Site Metal–Nitrogen–Carbon Catalysts. *Journal of the American Chemical Society* **2019,** *141* (31), 12372-12381. https://doi.org/10.1021/jacs.9b05576.

11. Li, X.; Wang, X.; Xiao, G.; Zhu, Y., Identifying active sites of boron, nitrogen co-doped carbon materials for the oxygen reduction reaction to hydrogen peroxide. *Journal of Colloid and Interface Science* **2021,** *602*, 799-809. https://www.sciencedirect.com/science/article/pii/S0021979721009425.

12. Byeon, A.; Cho, J.; Kim, J. M.; Chae, K. H.; Park, H.-Y.; Hong, S. W.; Ham, H. C.; Lee, S. W.; Yoon, K. R.; Kim, J. Y., High-yield electrochemical hydrogen peroxide production from an enhanced two-electron oxygen reduction pathway by mesoporous nitrogen-doped carbon and manganese hybrid electrocatalysts. *Nanoscale Horizons* **2020,** *5* (5), 832-838. http://dx.doi.org/10.1039/C9NH00783K.

13. Jung, E.; Shin, H.; Hooch Antink, W.; Sung, Y.-E.; Hyeon, T., Recent Advances in Electrochemical Oxygen Reduction to H2O2: Catalyst and Cell Design. *ACS Energy Letters* **2020,** *5* (6), 1881-1892. https://doi.org/10.1021/acsenergylett.0c00812.
